# On Casimir and Helmholtz Fluctuation-Induced Forces in Micro- and Nano-Systems: Survey of Some Basic Results

**DOI:** 10.3390/e26060499

**Published:** 2024-06-07

**Authors:** Daniel Dantchev

**Affiliations:** 1 Institute of Mechanics, Bulgarian Academy of Sciences, Academic Georgy Bonchev St., Building 4, 1113 Sofia, Bulgaria; danieldantchev@gmail.com; 2 Max-Planck-Institut für Intelligente Systeme, Heisenbergstrasse 3, D-70569 Stuttgart, Germany

**Keywords:** Casimir force, Helmholtz force, low-dimensional systems, phase transitions, critical phenomena, fluctuation-induced forces, finite-size scaling

## Abstract

Fluctuations are omnipresent; they exist in any matter, due either to its quantum nature or to its nonzero temperature. In the current review, we briefly cover the quantum electrodynamic Casimir (QED) force as well as the critical Casimir (CC) and Helmholtz (HF) forces. In the QED case, the medium is usually a vacuum and the massless excitations are photons, while in the CC and HF cases the medium is usually a critical or correlated fluid and the fluctuations of the order parameter are the cause of the force between the macroscopic or mesoscopic bodies immersed in it. We discuss the importance of the presented results for nanotechnology, especially for devising and assembling micro- or nano-scale systems. Several important problems for nanotechnology following from the currently available experimental findings are spelled out, and possible strategies for overcoming them are sketched. Regarding the example of HF, we explicitly demonstrate that when a given integral quantity characterizing the fluid is conserved, it has an essential influence on the behavior of the corresponding fluctuation-induced force.

## 1. Introduction

Let us consider two flat half-spaces *A* and *B* separated by a gap with thickness *L* filled with a medium *C* which fluctuates with the characteristic energy *E* of the pertinent fluctuations. If the fluctuations decay with the distance slowly enough, say, algebraically (which takes place if the corresponding excitations are massless), then changes in the fluctuations due to *A* will be felt by *B* (and vice versa), leading to a force between *A* and *B*. Such a force bears the natural name of fluctuation-induced force (FIF) $F^{\|}$, where ‖ simply reminds us about the geometry we are discussing. The *L*-dependence of $F^{\|}$ can be easily determined on dimensional grounds; as for any force, we have $F^{\|}\propto Energy/Length\propto E/L$. In the considered geometry, it is natural to consider the normalized force per unit area $A∼L^{2}$, then obtain it for the corresponding pressure $p^{\|}∼E/L^{3}$. Taking into account that in quantum systems $E∼h\nu_{0}∼hc/L$, where $\nu_{0}$ is some characteristic frequency of the quantum system, while in classical systems $E∼k_{B}T$, we obtain

(1) $$p_{\mathrm{quantum}}^{\|}∼hc/L^{4},\;p_{\mathrm{classical}}^{\|}∼k_{B}T/L^{3}.$$

Naturally, the classical result also holds if, in a given quantum system, $k_{B}T\gg h\nu_{0}$; in Equation (1), *h* is Planck’s constant, *c* is the speed of light, $k_{B}$ is Boltzmann’s constant, and *T* is the temperature of the system. Equation (1) reflects essential parts of the QED Casimir, critical Casimir (CCE), and (as we will see) Helmholtz force effects. Of course, the precise behavior of $p_{\mathrm{quantum}}^{\|}$ and $p_{\mathrm{classical}}^{\|}$, e.g., the corresponding pre-factors cannot be obtained in such a simple way, and the efforts of many scientists have been dedicated to figuring out the corresponding details. Below, we briefly comment on this topic.

**Figure 1:**
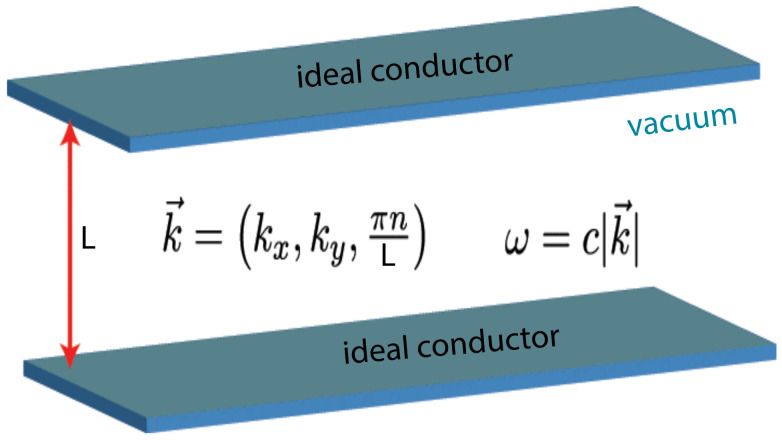
The setup of the system considered by Casimir in his original article [1].

The current most prominent example of a fluctuation-induced force involves the force due to quantum or thermal fluctuations of the electromagnetic field, leading to the so-called QED Casimir effect, named after the Dutch physicist H.B. Casimir. Casimir first realized that in the case of two perfectly-conducting, uncharged, and smooth plates parallel to each other in vacuum, at T=0 (see Figure 1) these fluctuations lead to an *attractive* force between them [1]. In other words, Casimir demonstrated that the boundary conditions imposed by two plates (denoted in the following by ∥) on the spectrum of the quantum mechanical zero-point fluctuations of the electromagnetic field lead to the above remarkable mechanical effect involving the appearance of a long-range *attractive* force between the plates. More precisely, for the corresponding pressure he obtained

(2) $$F_{\mathrm{Cas}}^{\|}\left(L\right)=-\frac{\pi^{2}}{240}\frac{\hbar c}{L^{4}}=-1.3\times 10^{-3}\frac{1}{{\left(L/\mu m\right)}^{4}}\frac{N}{m^{2}}.$$

Intuitively, this pressure can be viewed as the difference in radiation pressure of virtual photons outside and inside the pore formed by the two plates, which results in an attractive force $F_{\mathrm{Cas}}^{\|}$ between them [2]. The accepted terminology terms the negative force as the attractive one. In order to derive Equation (2), Casimir calculated the derivative with respect to *L* of the energy difference δE [1]: (i) when *A* and *B* are at infinite distance, and (ii) when they are at a distance *L* from each other (see below for more details).

There is a vast amount of literature concerning research on the quantum Casimir effect. Here, we only mention the review articles in [3,4,5,6,7,8,9,10,11,12,13,14,15,16,17,18,19,20,21,22,23,24,25,26,27,28,29,30,31,32,33,34,35,36,37,38], involving recent studies of the *dynamical* Casimir effect (in which actual photons can be created if a single mechanical mirror undergoes accelerated motion in vacuum) [39,40,41,42,43,44,45,46] and studies of the effects which emerge in systems out of thermodynamic equilibrium (in which the material bodies are characterized by different temperatures) [47,48,49,50,51,52,53]. Currently, the QED Casimir effect is a popular subject of research. The Casimir and Casimir-like effects are objects of studies in quantum electrodynamics, quantum chromodynamics, cosmology, condensed matter physics, and biology, with some elements of it present in nanotechnology as well. Investigations devoted to the topic are currently being performed on many fronts, including research ranging from attempts to unify the four fundamental forces of nature [6,11,12] to rather more practical issues such as the design and the performance of micro- and nanoscale machines [16,22,23,54,55]. Readers interested in these topics can consult the existing reviews cited above.

**Figure 2:**
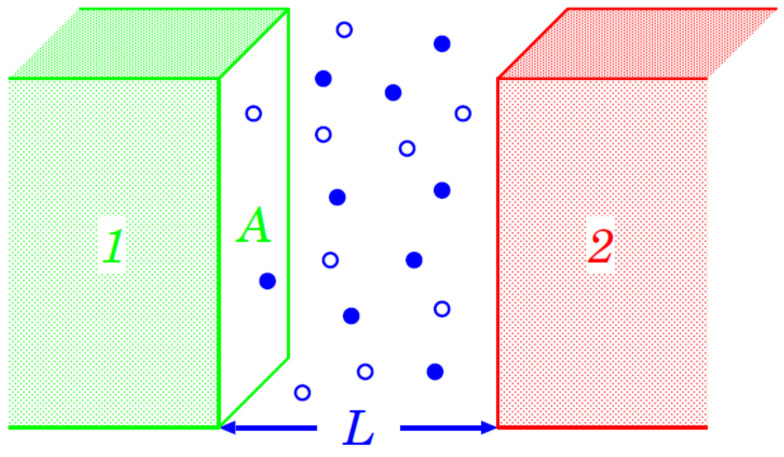
The basic setup for discussing the thermodynamic Casimir effect [56].

Looking at the problem from the historical perspective, in 1978, thirty years after Casimir, Fisher and De Gennes [56] showed that a very similar effect exists in fluids. As the main setup for discussing the CCE, we can envisage two material bodies, (1) and (2), immersed in a fluid (see Figure 2). They exert an effective force $F^{\left(1,2\right)}$ on each other, which is mediated by the fluid; this includes, inter alia, the direct interaction between material bodies (1) and (2). If the thermodynamic state of the fluid is far away from a bulk phase transition at $T=T_{c}$, this force varies slowly and smoothly as function of the temperature *T*. Upon approaching $T_{c}$ of a continuous phase transition, $F^{\left(1,2\right)}$ acquires a contribution $F_{\mathrm{Cas}}^{\left(1,2\right)}$ due to the critical fluctuations of the confined fluid. This singular contribution encompasses both the distortion of the local (eventually) nonzero order parameter (due to the finite distance between (1) and (2)) and the fluctuations of the order parameter. The singular contribution $F_{\mathrm{Cas}}^{\left(1,2\right)}$ follows by subtracting the smooth background contribution (after extrapolating it to the neighborhood of $T_{c}$) from $F^{\left(1,2\right)}$. Upon the above construction, in the disordered phase, $F^{\left(1,2\right)}$ and $F_{\mathrm{Cas}}^{\left(1,2\right)}$ vanish in the limit of increasing separation between the bodies (1) and (2). Taking into account that in the *d*-dimensional space the surface $A\propto L^{d-1}$ and that the energy of the fluctuations $E\propto k_{B}T$, it can be concluded that $F_{\mathrm{Cas}}^{\|}\propto k_{B}T/L^{d}$ for the thermodynamic Casimir effect near the critical point of the system. For a (d=3)-dimensional system, we can write the force at the critical point $T=T_{c}$ in the following form:

(3) $$F_{\mathrm{Cas}}^{\left\|(\tau \right)}\left(T=T_{c},L\right)≃8.1\times 10^{-3}\frac{\Delta^{\left(\tau \right)}\left(d=3\right)}{{\left(L/\mu m\right)}^{3}}\frac{T_{c}}{T_{\mathrm{roon}}}\frac{N}{m^{2}}$$

where $T_{\mathrm{room}}$ = 20 °C (293.15 K). The above equation is in a full agreement with Equation (1). In order to calculate $F_{\mathrm{Cas}}^{\left\|(\tau \right)}$, it is normally necessary to determine the finite size-dependent part of the properly normalized difference between the energy of the infinite and finite systems, similar to the QED case (see below). Here, $\Delta^{\left(\tau \right)}$ is the so-called *Casimir amplitude*, which depends on the bulk and surface *universality classes* (see below) of the system and the applied *boundary conditions*τ. For most systems and boundary conditions, $\Delta^{\left(\tau \right)}\left(d\right)=O\left(1\right)$; thus, when $T_{c}≃T_{\mathrm{room}}$, both forces (quantum and thermodynamic) can be of the same order of magnitude, i.e., they can both be essential, measurable, and obviously significant at or below the micrometer length scale. We stress here that $\Delta^{\left(\tau \right)}\left(d\right)$ can be both *positive and negative*, i.e., $F_{\mathrm{Cas}}^{\left\|(\tau \right)}\left(T,L\right)$ can be both *attractive and repulsive*. Recently, a review of the exact results available for the CCE has been published [57]; overviews of different aspects of this effect can be found in [57,58,59,60,61].

The above arguments for two semi-spaces *A* and *B* separated by a fluid *C* can be easily extended for bodies *A* and *B* of general shape *immersed* in a fluid, say, two spheres, a sphere and a semi-space (plane surface), etc. In the general case, it is the confinement of a fluctuating field by the surfaces of the material bodies which causes FIF acting on the confining surfaces of these bodies. In the current review, we do not consider such geometries, and simply refer interested readers to the existing reviews on QED and the critical Casimir effects for further details.

When considering the FIF between *A* and *B* immersed in a fluctuating medium *C*, it is always supposed that the constituents of *C* can enter and leave the region between objects *A* and *B*. There are, however, two important subcases. In the first, *C* is in contact with a reservoir, i.e., its constituents can enter and leave the part of the space occupied by *A* and *B*. In this case, we can speaks of the bona fide Casimir force. In the second case, the system itself is bounded such that some integral quantity characterizing the amount of material in *C* is *conserved*, say, the total mass or the integral over the volume of the system or the order parameter. In this case, we can speak about the recently introduced (see [62]) and not yet well studied *Helmholtz force*. Currently, there are only a few articles devoted to these forces [62,63,64,65]. Note that in HF case we are again near a critical point of the medium *C*. As it turns out, however, the HF has a behavior distinctly different from that of the Casimir force. Because the study of Helmholtz forces is a relatively new field of research, in the current review we devote special attention to it and its behavior, both as a function of the temperature and of an external ordering field characterizing the medium *C*. We show this via exact results for the one-dimensional Ising model in a fixed-order parameter *M* ensemble. We stress that in customarily considered applications involving, say, the equilibrium Ising model with respect to binary alloys or binary liquids, if one insists on full rigor, the case with a fixed order parameter must be addressed. It is interesting to note that the HF in the case of periodic boundary conditions shows behavior similar to that appearing in certain versions of the big bang theory, e.g., strong repulsion at high temperatures transitioning to moderate attraction for intermediate values of the temperature and then back to repulsion, albeit much more weakly than during the initial period of highest temperature.

We stress that the definition and existence of Helmholtz force (see Section 4) is by no means limited to the Ising chain, and can be addressed in principle in any model of interest.

In addition, we note that the issue of the ensemble dependence of fluctuation-induced forces pertinent to the ensemble has yet to be studied.

The remainder of this review is structured as follows. First, in Section 2 we briefly consider the QED Casimir effect. Section 3 introduces details of the critical and thermodynamic Casimir effects, while Section 4 presents the definition of the Helmholtz force. Results for this force using the example of Ising chains with periodic, antiperiodic, and Dirichlet boundary conditions as well as with a defect bond are summarized in Section 5. Finally, concluding remarks about the FIF, including their role in nanotechnology, are presented in Section 6.

## 2. The QED Casimir Effect

As already alluded to in the introduction, the confinement of a fluctuating field generates effective forces on the confining surfaces, which nowadays are termed FIF.

The relationship in Equation (2) can be derived by considering the change of the structure of the electromagnetic modes between the two plates as compared with those in free space after assigning the zero-point energy $\frac{1}{2}\hbar \omega$ to each electromagnetic mode, i.e., photon of frequency ω. We emphasize that, in the absence of charges on the plates, the mean value of the electric field E and the magnetic field B vanishes, i.e.,

(4) 〈E〉=0and〈B〉=0,

while

(5) $$\langle E^{2}\rangle \neq 0\;\mathrm{and}\;\langle B^{2}\rangle \neq 0,$$

meaning that the expectation value of the energy due to the electromagnetic field, i.e., 〈H〉 with

(6) $$H=\int \left[\frac{1}{2}\varepsilon_{0}E^{2}\left(r\right)+\frac{1}{2\mu_{0}}B^{2}\left(r\right)\right]\;d^{3}r,$$

is nonzero. In fact, according to quantum field theory, the energy of the electromagnetic field in the vacuum state in free space is infinitely large, and all physically relevant energies are measured relative to the energy of the vacuum. When the two (in the above sense ideal) parallel smooth metal plates are placed against each other in free space, the tangential component of the electric field and the normal component of the magnetic induction must vanish at the plate surfaces. As a result, not all zero-point oscillations occur. Subtracting the energy of the vacuum energy in free space from that of the allowed modes, after taking the derivative with respect to *L*, we obtain the result reported in Equation (2). In more detail, focusing on a single component of the electric field, which vanishes at the surface of the metal (this discussion can be easily extended to include all vector components), the simultaneous presence of two parallel walls restricts the allowed wave vector $k_{⊥}$ of the field in the direction normal to the plates, meaning that the field itself vanishes at both walls (Dirichlet boundary conditions). If we instead impose no restriction on the wave vector $k_{∥}$ parallel to the plates, which are assumed to have a large lateral extension with a transverse area *A* and to be separated by a distance *L*, then we have $k_{⊥}=\pi n/L$ with n=0,1,2,⋯, and the energy E contained within the confined space takes the form

(7) $$E=\sum_{k}\frac{1}{2}\hbar \omega \left(k\right),$$

where the sum runs over all allowed values of $k=(k_{∥},k_{⊥})$ and where $\omega \left(k\right)\equiv \omega_{k}=c\left|k\right|=c\sqrt{k_{∥}^{2}+k_{⊥}^{2}}$ (with |k|=k) is the frequency of the mode with wave vector k. As it stands, the sum in Equation (7) diverges due to the fact that $|\omega_{k}|$ grows as *k* increases and because the sum lacks any ultraviolet cutoff, i.e., *k* is allowed to grow unboundedly. In this context, we are actually interested in the energy of the modes which can be “contained” by the metallic cavity, as they are the only ones affected by the presence of the cavity itself. At sufficiently high frequencies, the cavity becomes transparent to the electromagnetic field. Note that within the assumptions made when deriving Equation (2), no materials-dependent properties enter the picture; thus, the force depends only on the Planck constant *ℏ* and the speed of light in vacuum *c* as well as on the geometry of the pore (characterized here by *L*). In addition, the force does not depend on the electric charge *e*, implying that, for the present conditions of the phenomenon, the coupling between the electromagnetic field and the matter is unimportant, as is any other interaction.

In the aftermath of [1], there has been an intense theoretical effort to describe the force beyond the case of ideal plates by considering the actual dielectric properties of the two plates and the medium in between [17,66,67,68,69,70,71,72,73,74,75,76]. Specifically, the groundbreaking results of Lifshitz et al. [66,77], who developed a unified theory of the van der Waals and the Casimir forces, must be mentioned. It should be noted that, when discussing the quantum Casimir effect, the retarded van der Waals interactions are often called Casimir interactions; however, we retain the notion of retarded van der Waals interactions when discussing the thermodynamic Casimir effect in order to avoid confusion of these forces with the critical Casimir force, which we discuss later.

Lifshitz et al. studied the case of two materials acting as walls, described by frequency-dependent dielectric permittivities $\varepsilon^{\left(n\right)}\left(\omega \right),n=1,2$, separated by a third material characterized by $\varepsilon^{\left(0\right)}\left(\omega \right)$. It turns out that in the limit of small separations (still large compared with molecular scales), the Casimir force approaches the more familiar van der Waals force [17,78]. This more realistic description provides specific predictions which are amenable to high-precision measurements. The corresponding expressions for the force are also quite instructive in that they allows for prediction of how the material properties of the substances involved have to be tuned in order to modify the strength (and even the sign) of the force. According to Lifshitz, in order to calculate the Casimir pressure for a set of three dielectric materials, it is necessary to know their permittivities along the imaginary frequency axis. As experimental data for the complex permittivity

(8) $$\varepsilon \left(\omega \right)=\varepsilon^{\prime }\left(\omega \right)+i\varepsilon^{\prime \prime }\left(\omega \right),$$

where $\varepsilon^{\prime },\varepsilon^{\prime \prime }\in R$, exist only for real frequencies, the permittivity along the imaginary frequency axis has to be determined from the Kramers–Kronig relation (see, e.g., [79]):

(9) $$\varepsilon \left(i\xi \right)=1+\frac{2}{\pi }\int_{0}^{\infty }\frac{x\varepsilon^{\prime \prime }\left(x\right)}{x^{2}+\xi^{2}}dx.$$

From the Lifshitz theory, it can be inferred [78] that it is possible to observe Casimir *repulsion* in the film geometry if the two half-spaces (1) and (2) forming the plates and confining the film (0) exhibit permittivities which fulfill the relationship

(10) $$\varepsilon^{\left(2\right)}\left(i\xi \right)<\varepsilon^{\left(0\right)}\left(i\xi \right)<\varepsilon^{\left(1\right)}\left(i\xi \right).$$

Experimentally, repulsion occurs if the inequality in Equation (10) holds over a *sufficiently wide frequency range*. Actually, this is a widespread phenomenon shared by all substrate–fluid systems which show complete wetting, such as in the old experiment by Sabisky and Anderson [80]. Accordingly, Casimir repulsion is a common feature [81]. In fact, it has been already observed; see, e.g., [82].

A standard approximation for obtaining the force between two bodies of nontrivial shape is the so-called Derjaguin approximation (DA) [83]. The DA term is used in colloidal science (see, e.g., [84] and p. 34 in [85]), while it is known as proximity force approximation in studies of the QED Casimir effect (see, e.g., p. 97 in [17]). The main idea behind the DA is that it is possible to relate the knowledge of the interaction force/potential between two parallel plates with that between two gently curved colloidal particles when the separation between them is much smaller than the geometrical characteristics of the particles in question. More specifically, the DA states that in d=3, the interaction force $F^{R_{1},R_{2}}\left(L\right)$ between two spherical particles with radii $R_{1}$ and $R_{2}$ placed at a distance $L\ll R_{1},R_{2}$ is provided by

(11) $$F_{\mathrm{DA}}^{R_{1},R_{2}}\left(L\right)=2\pi R_{\mathrm{eff}}\int_{L}^{\infty }f_{A}^{\|}\left(z\right)dz,$$

where $R_{\mathrm{eff}}^{-1}=R_{1}^{-1}+R_{2}^{-1}$ is an effective radius and $f_{A}^{\|}$ is the force per unit area between parallel plates. When a sphere with radius $R_{1}\equiv R$ interacts with a plate, we have $R_{2}=\infty$. in which case (11) is still valid with $R_{\mathrm{eff}}=R$.

An improvement and generalization of the DA called the “surface integration approach” (SIA) has been proposed in [86]. It has been used there to study van der Waals interactions between objects of arbitrary shape and a plate of arbitrary thickness. It delivers *exact* results if the interactions involved can be described by *pair potentials*. The main advantage of this approach over the DA is that one is no longer bound by the restriction that the interacting objects must be much closer to each other than their characteristic sizes. The main result is that for the force acting between a 3d object (say a colloid particle) B≡{(x,y,z),(x,y,z)∈B} of general shape S(x,y)=z and a flat surface bounded by the (x,y)—plane of a Cartesian coordinate system, we have

(12) $$\begin{array}{c} F_{\mathrm{SIA}}^{B,|}\left(L\right)=\int_{A_{S}^{\mathrm{to}}}\int f_{A}^{\|}\left[S\left(x,y\right)\right]dxdy-\int_{A_{S}^{\mathrm{away}}}\int f_{A}^{\|}\left[S\left(x,y\right)\right]dxdy, \end{array}$$

where $A_{S}$ is the projection of the surface *S* of the particle over the (x,y)− plane, with $A_{S}=A_{S}^{\mathrm{to}}⋃A_{S}^{\mathrm{away}}$. Equation (12) has a very simple and intuitive meaning: in order to determine the force acting on the particle, it is necessary to subtract from the contributions stemming from surface regions $A_{S}^{\mathrm{to}}$ that “face towards” the projection plane those from regions $A_{S}^{\mathrm{away}}$ that “face away” from it, where $A_{S}^{\mathrm{to}}$ and $A_{S}^{\mathrm{away}}$ are the projections of the corresponding parts of the surface of the body on the (x,y)—plane. It is clear that when taking into account only the contributions over $A_{S}^{\mathrm{to}}$, we obtain expression very similar to those of DA. In this case, the two expressions differ only in the fact that (12) takes into account that the force on a given point of the *S* is *along the normal* to the surface at that point, while the standard DA does not take this into account. Recall that (12) provides exact results for the interaction under the assumption that the constituents of the body interact via pair potentials. This is, strictly speaking, *not* the case of the force in QED Casimir, i.e., Equation (12) is still an approximation. It is, however, clear that under mechanical equilibrium of the colloid in the fluid, the force is indeed along the normal to the surface at the point of the surface where it acts. Thus, it is possible to obtain a reasonably good approximation of the effect of this force by keeping only the integration over parts of the surface of the body that faces the plane. It should be noted that the importance of the SIA approach has been already recognized and used; see, e.g., [87,88,89,90,91,92]. For the QED Casimir force, such a generalization has been derived on the solid basis of quantum field theory in the framework of scattering approaches (see, e.g., [93,94]). This approach is widely used in making comparisons between theory and measurements of the QED Casimir force in sphere–plate geometries (see, e.g., [95]).

## 3. The Critical and Thermodynamic Casimir Effects

As explained above, the spatial restriction on the fluctuations of the order parameter describing a continuous phase transition of a many-body system leads to the so-called *critical Casimir effect* [56]. Then, the interactions in the system are mediated not by photons, as in the case of the electromagnetic field, but by different types of massless excitations. In the case that the critical point has a quantum origin, and instead of temperature certain quantum parameters govern the fluctuations in the system, we speak of the *quantum critical Casimir effect* [59,96]. In addition, systems such as liquid ^4^He and liquid crystals, i.e., so-called correlated fluids, exhibit gapless excitations called Goldstone modes [8,97,98,99]. These fluctuations also lead to long-ranged forces between the boundaries of the systems, although such systems are thermodynamically positioned below their respective critical points. For these cases, we speak of the noncritical Casimir effect, or more generally the *thermodynamic Casimir effect*. We shall use the latter notion as a general one that encompasses all cases in which the Casimir effect is due to the fluctuations of a certain order parameter.

The critical Casimir effect depends on the parameters describing the thermodynamic state of the critical medium, such as the temperature and an externally applied field (e.g., pressure, excess chemical potential, magnetic field), as well as on the distance *L* between (1) and (2); that is, the observed phenomenon is a *finite size effect*. Therefore, if *L* increases the effect, the magnitude of the associated force decreases and eventually vanishes.

Any thermodynamic system which is of finite extent in *at least one* spatial direction is called a *finite-size system*. The corresponding modification of its phase behavior compared with that of the bulk is described by *finite-size scaling theory* [59,100,101]. Because of the deep interconnection between the theory of the thermodynamic Casimir effect and finite-size scaling theory, we recall some basic facts concerning finite-size scaling theory which are relevant for studying the thermodynamic Casimir effect. We start by recollecting some basic properties of critical phenomena in bulk systems. In the vicinity of the bulk critical point $\left(T_{c},h=0\right)$ governed by the temperature *T* and some external field *h*, the bulk correlation length of the order parameter ξ becomes large and theoretically diverges: $\xi_{t}^{+}\equiv \xi \left(T\to T_{c}^{+},h=0\right)≃\xi_{0}^{+}t^{-\nu }$, $t=\left(T-T_{c}\right)/T_{c}$ and $\xi_{h}\equiv \xi \left(T=T_{c},h\to 0\right)≃\xi_{0,h}{\left|h/\left(k_{B}T_{c}\right)\right|}^{-\nu /\Delta }$, where ν and Δ are the usual critical exponents and $\xi_{0}^{+}$ and $\xi_{0,h}$ are the corresponding nonuniversal amplitudes of the correlation length along the *t* and *h* axes. If, in a finite system, ξ becomes comparable to *L*, the thermodynamic functions describing its behavior depend on the ratio L/ξ and take scaling forms provided by finite-size scaling theory. Further information of the phase transitions and related physical and mathematical problems can be found in [59,100,101] and in the set of articles on the topic cited therein.

Crucial by its implications for systems with continuous phase transitions is the so-called *universality hypothesis*. It was initially formulated by Kadanoff [102]. According to this hypothesis, “all (continuous) phase transition problems can be divided into a small number of different classes depending upon the dimensionality of the system and the symmetries of the ordered state. Within each class, all phase transitions have identical behavior in the critical region; only the names of thermodynamic variables are changed”. All such systems are then part of the same *universality class*. For example, we have the Ising universality class characterized by breaking of the $Z_{2}$ symmetry of the original effective Hamiltonian for the scalar order parameter, the XY universality class with a two-component order parameter and a disordered phase with O(2) symmetry, and the Heisenberg universality class characterized by a vectorial order parameter with an O(3) symmetry. Any of these bulk universality classes is accompanied by a set of surface universality classes which depend on the behavior of the order parameter near and at a surface(s) of the semi-infinite or finite system. For a film geometry, the accumulated experimental and theoretical evidence supports the statement that the Casimir force is attractive when the boundary conditions on either plate are the same or similar and is repulsive when they essentially differ from each other. For the case of a one-component fluid, the latter means that one of the surfaces adsorbs the liquid phase of the fluid while the other prefers the vapor phase. This rule, which connects the type of boundary condition with the type of the force, seems to be violated if competing interactions are present in the system. Recently, it has been established that, under periodic boundary conditions, an Ising chain with a defect bound can have a Casimir force which changes sign as a function of *T* provided that an antiferromagnetic bond is present within the otherwise ferromagnetic bonds of the chain [65].

In the remainder of the current section, we discuss the thermodynamic Casimir effect in a system with a $\infty^{d-1}\times L$ film geometry. We envisage a system exposed to a temperature *T* and an external ordering field *h* that couples to its order parameter, i.e., density, concentration difference, magnetization *M*, etc. We imagine as examples a simple fluid system at its liquid–vapor critical point, a magnet at the phase transition from paramagnetic to ferromagnetic state, and a binary liquid mixture or binary alloy with phases *A* and *B* near its consolute temperature point. Letting $\left(T=T_{c},h=0\right)$ be this bulk critical point in the (T,h) plane, we consider only the case of an one-dimensional order parameter ϕ∈R. The thermodynamic Casimir force $F_{\mathrm{Cas}}\left(T,h,L\right)$ in such a system is the *excess pressure* over the bulk pressure, and acts on the boundaries of the finite system due to the finite size of the system, i.e.,

(13) $$F_{\mathrm{Cas}}\left(T,h,L\right)=P_{L}\left(T,h\right)-P_{b}\left(T,h\right),$$

where $P_{L}$ is the pressure in the finite system while $P_{b}$ is that in the infinite system. Note that the above definition is actually equivalent to another commonly used definition [58,59,103]:

(14) $$F_{\mathrm{Cas}}\left(T,h,L\right)\equiv -\frac{\partial \omega_{\mathrm{ex}}\left(T,h,L\right)}{\partial L}=-\frac{\partial \omega_{L}\left(T,h,L\right)}{\partial L}-P_{b}$$

where $\omega_{\mathrm{ex}}=\omega_{L}-L\;\omega_{b}$ is the excess grand potential per unit area, $\omega_{L}$ is the grand canonical potential of the finite system, again per unit area, and $\omega_{b}$ is the density of the grand potential for the infinite system. The equivalence between the definitions in Equations (13) and (14) comes from the observation that $\omega_{b}=-P_{b}$ and that for a finite system with surface area *A* and thickness *L* it is the case that $\omega_{L}=\lim_{A\to \infty }\Omega_{L}/A$ with $-\partial \omega_{L}\left(T,h,L\right)/\partial L=P_{L}$. When $F_{\mathrm{Cas}}\left(\tau ,h,L\right)<0$, the excess pressure of the system will be inward; this corresponds to an *attraction* of the surfaces of the system towards each other, and conversely to a *repulsion* if $F_{\mathrm{Cas}}\left(\tau ,h,L\right)>0$. Then, for a system positioned near its critical point, the finite-size scaling theory [58,59,100,101,104,105,106] predicts for the CF that

(15) $$F_{\mathrm{Cas}}\left(t,h,L\right)=L^{-d}X_{\mathrm{Cas}}\left(x_{t},x_{h}\right),$$

where $x_{t}=a_{t}tL^{1/\nu }$, $x_{h}=a_{h}hL^{\Delta /\nu }$. In Equation (15), *d* is the dimension of the system, while $a_{t}$ and $a_{h}$ are nonuniversal metric factors that can be fixed for a given system by taking them to be, e.g., $a_{t}=1/{\left[\xi_{0}^{+}\right]}^{1/\nu }$ and $a_{h}=1/{\left[\xi_{0,h}\right]}^{\Delta /\nu }$, respectively.

## 4. Casimir Force versus Helmholtz Force

When the degrees of freedom can freely enter and leave the medium *C*, then we speak of the *Casimir force*. Within the realm of statistical mechanics, such a force is described within the grand canonical ensemble (GCE). In a recent Letter [62] (and see [63,64,65]), we have introduced the term of a *Helmholtz* FIF. This is a force in which an integral quantity characterizing the medium *C* is fixed. When this conserved quantity is the total order parameter value, the system and the corresponding forces are described within the canonical ensemble (CE). In the current section, we show that the Casimir force and the Helmholtz force have substantially different behaviors for one and the same system and for the same boundary conditions. We stress that the issue of ensemble dependence of critical fluctuation-induced forces is not yet well explored, and only few articles devoted to it are available in the literature.

### 4.1. Casimir Force and Grand Canonical Ensemble

We consider a finite lattice $L\in Z^{d}$ with each site r on the *d*-dimensional lattice embedded with a spin variable $s_{r}\in R^{n}$. The spins interact via exchange interactions $J(r,r^{\prime })$. The corresponding Hamiltonian is

(16) $$H\left(\left\{s_{i}\right\}\right)=-\sum_{r,r^{\prime }\in L}J\left(r,r^{\prime }\right)\;s_{r}\cdot s_{r^{\prime }}.$$

In the simplest possible case, the spins interact through a nearest neighbor exchange interaction *J*. Then, the sum runs over the nearest neighbor pairs $\langle r,r^{\prime }\rangle$ on the lattice. For the simplicity of the notations, in the remainder we concentrate on only such interactions unless explicitly stated otherwise. For n=1, we can speak of Ising-type models, for n=2 of XY-type models, and for n=3 of Heisenberg-type models. In the simplest Ising-type model, the spins can only take values of +1 and −1.

In statistical mechanics, the systems are described within so-called Gibbs ensembles of dependent random variables. More specifically, in the grand canonical ensemble, i.e., in the presence of an external bulk field *h*, the Hamiltonian of an Ising type model is

(17) $$H_{\mathrm{GCE}}\left(\left\{s_{r}\right\},h\right)=H\left(\left\{s_{r}\right\}\right)-h\sum_{r}s_{r}.$$

Then, the partition function of the grand canonical ensemble of a system with *N* particles is

(18) $$Z_{\mathrm{GCE}}\left(N,\beta ,h\right)=\sum_{\left\{s_{r}\right\}}\exp\left[-\beta H_{\mathrm{GCE}}\left(\left\{s_{r}\right\},h\right)\right],\;\mathrm{where}\;\beta =1/\left(k_{B}T\right).$$

Obviously, for the total average magnetization $\bar{M}$, we have

(19) $$\bar{M}\equiv \langle \sum_{r}s_{r}\rangle =\frac{\partial }{\partial (\beta h)}\ln\left[Z_{\mathrm{GCE}}\left(N,\beta ,h\right)\right].$$

Within the grand canonical ensemble, the corresponding fluctuation-induced force is called the thermodynamic Casimir force; its definition follows below.

### 4.2. Definition of the Thermodynamic Casimir Force

When the partition function in Equation (18) is known, it is possible to determine the total Gibbs free energy $F_{\mathrm{tot}}$ via

(20) $$\beta F_{\mathrm{tot}}\left(N,\beta ,h\right)=-\ln\left[Z_{\mathrm{GCE}}\left(N,\beta ,h\right)\right]$$

and the Casimir force [57]

(21) $$\beta F_{\mathrm{Cas}}^{\left(\zeta \right)}\left(T,h,L\right)\equiv -\frac{\partial }{\partial L}f_{\mathrm{ex}}^{\left(\zeta \right)}\left(T,h,L\right),$$

where

(22) $$f_{\mathrm{ex}}^{\left(\zeta \right)}\left(T,h,L\right)\equiv f^{\left(\zeta \right)}\left(T,h,L\right)-Lf_{b}\left(T,h\right)$$

is the so-called excess over the density contribution of the bulk free energy $Lf_{b}\left(T,h\right)$ normalized per area and per $k_{B}T$. Here, a system is envisaged in a film geometry $\infty^{d-1}\times L$, $L\equiv L_{⊥}$, with boundary conditions ζ imposed along the spatial direction of finite extent *L* and with total free energy $F_{\mathrm{tot}}$, while $f^{\left(\zeta \right)}\left(T,h,L\right)\equiv \lim_{A\to \infty }F_{\mathrm{tot}}/A$ is the free energy per area *A* of the system.

### 4.3. Helmholtz Force and Canonical Ensemble

Within the canonical Gibbs ensemble, the Hamiltonian of the model is

(23) $$H_{\mathrm{CE}}\left(\left\{s_{r}\right\}\right)=H\left(\left\{s_{r}\right\}\right)\;\;\mathrm{with}\;\mathrm{the}\;\mathrm{constraint}\;\sum_{r}s_{r}=M,$$

i.e., only configurations with a given fixed value of *M* are allowed. The statistical sum within this ensemble is then

(24) $$Z_{\mathrm{CE}}\left(N,\beta ,M\right)=\sum_{\left\{s_{r}\right\},\sum_{i=1}^{N}s_{r}=M}\exp\left[-\beta H_{\mathrm{CE}}\left(\left\{s_{r}\right\}\right)\right],\;\beta =1/\left(k_{B}T\right).$$

Within the canonical ensemble, the corresponding fluctuation-induced force is called the Helmholtz force [62,63,64,65]. Its definition follows below.

### 4.4. On the Definition of the Helmholtz Force

From $Z_{\mathrm{CE}}\left(N,\beta ,M\right)$, according to the principles of the statistical mechanics, it is possible to determine the total Helmholtz free energy

(25) $$\beta A_{\mathrm{tot}}=-\ln Z_{\mathrm{CE}}\left(N,\beta ,M\right),$$

which allows for the determination of a fluctuation-induced force $F_{H}\left(T,M,L\right)$ in the fixed *M*-ensemble, i.e., in the T−M ensemble, which we call the *Helmholtz force*. This can be achieved in a manner similar to the definition of the Casimir force for critical systems in the grand canonical *T*–*h* ensemble. Along these, lines we define

(26) $$\beta F_{H}^{\left(\zeta \right)}\left(T,M,L\right)\equiv -\frac{\partial }{\partial L}a_{\mathrm{ex}}^{\left(\zeta \right)}\left(T,M,L\right)$$

where

(27) $$a_{\mathrm{ex}}^{\left(\zeta \right)}\left(T,M,L\right)\equiv La_{H}^{\left(\zeta \right)}\left(T,M,L\right)-L\;a_{H}\left(T,m\right),$$

with $m=\left[\lim_{A\to \infty }\left(M/A\right)\right]/L$; here, $a_{H}\left(T,M,L\right)\equiv \left[\lim_{A\to \infty }A_{\mathrm{tot}}/A\right]/L$ is the Helmholtz free energy density of the finite system and $a_{H}\left(T,m\right)$ is that of the infinite system.

In [62,63,64,65], it was shown that the *Helmholtz fluctuation-induced force* defined in this way shows behavior that is very different from that of the Casimir force. More specifically, in [62] it was demonstrated that for an Ising chain with fixed *M* under periodic boundary conditions, $F_{H}^{\left(\mathrm{per}\right)}\left(T,M,L\right)$ can be attractive or repulsive depending on the temperature *T*, while $F_{\mathrm{Cas}}^{\left(\mathrm{per}\right)}\left(T,h,L\right)$ can only be attractive. As stated above, the issue of the ensemble dependence of fluctuation-induced forces pertinent to the ensemble has not, to the best of our knowledge, been studied yet in a thorough way. We stress that this issue is by no means limited to Ising chains, and can in principle be addressed in *any* model of interest. This issue can also be viewed as a useful addition to approaches to FIF in the fixed-*M* ensemble based on Ginzburg–Landau–Wilson Hamiltonians [107,108,109], in which context the usual *Casimir force* would be studied.

## 5. Some Results for the Helmholtz Force versus the Casimir Force

Following [62,63,64,65], we report below some exact results for the Helmholtz and Casimir forces for a one-dimensional Ising model with periodic, antiperiodic, and Dirichlet boundary conditions as well as for the more general case of a a chain with a defect bond. More precisely, we consider a one-dimensional Ising chain of *N* spins $\left(S_{i}\pm 1,i=1,\ldots ,N\right)$ with interaction *J* between them of a ferromagnetic type, i.e., J>0. The Hamiltonian of the model is provided by

(28) $$H^{\left(\zeta \right)}=-J\sum_{i=1}^{N-1}S_{i}S_{i+1}-J_{BC}S_{1}S_{N}+h\sum_{i=1}^{N}S_{i}.$$

**Figure 3:**
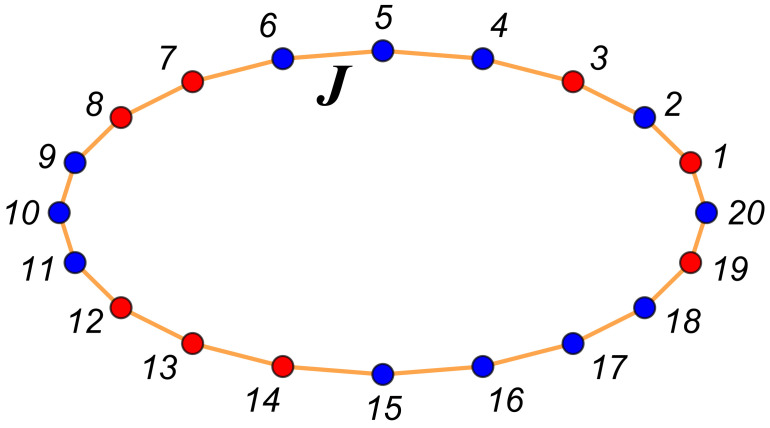
One-dimensional Ising model chain in a ring form. This is equivalent to a system with periodic boundary conditions. In the considered example, M=4, i.e., the number of “blue” atoms (molecules) is with 4 more than the number of “red” ones. It is also possible to consider that, say, the blue dots represent spins “up”, i.e., $s_{i}=+1$, while the red ones represents spins “down”, i.e., $s_{i}=-1$.

**Figure 4:**
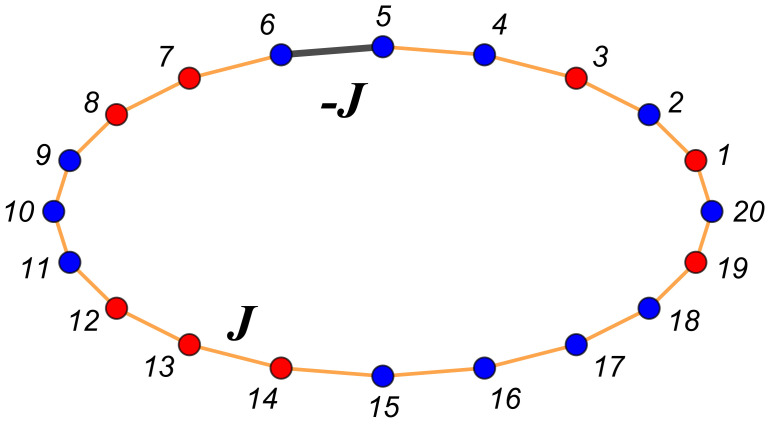
One-dimensional Ising model chain in a ring form and one opposite (or defect) bond. This is equivalent to a system with antiperiodic boundary conditions. In the considered example, M=4, i.e., the number of “blue” atoms (molecules) is 4 more than the number of “red” ones. As in the periodic case, it is possible to consider the blue dots as depicting spins “up”, i.e., $s_{i}=+1$, and red ones as representing spins “down”, i.e., $s_{i}=-1$.

When $J_{BC}=-J,J,0,J_{a}$, our boundary conditions are periodic (PBCs, ζ≡per), antiperiodic (ABCs, ζ≡anti), or Dirichlet–Dirichlet (DBCs, ζ≡D, also termed free or missing neighbors); when $J_{BC}=J_{a}$, where $J_{a}$ can have both positive or negative values, we term the case a *model with a defect bond*. In this last case we use the notation ζ=db. Let K=βJ, $K_{a}=\beta J_{a}$ and $\beta \equiv 1/(k_{B}T)$; examples of possible configurations with N=20 and M=4 are shown in Figure 3 for the case of periodic boundary conditions and in Figure 4 for antiperiodic ones.

The results for both the partition function and the force depend on the ensemble and the boundary conditions. For the corresponding partition functions, we have the following:

(i) For *periodic* boundary conditions:

Z(per)(N,K,M)=NeK(N−4)F1212(−M−N+2),12(M−N+2);2;e−4K,

where F12(α,β;γ;z) is the generalized hypergeometric function [110].

(ii) For the *Dirichlet* (missing neighbors at both ends of the chain) boundary conditions:

(29) Z(D)(N,K,M)=eK(N−1)[2e−2KF1212(−M−N+2),12(M−N+2);1;e−4K−12e−4K(M−N+2)F1212(−M−N+2),12(M−N+4);2;e−4K+12e−4K(M+N−2)F1212(−M−N+4),12(M−N+2);2;e−4K].

(iii) For the *antiperiodic* boundary conditions:

(30) Z(anti)(N,K,M)=eK(N−6)2e4K−1F1212(−M−N+2),12(M−N+2);1;e−4K+NF1212(−M−N+2),12(M−N+2);2;e−4K.

(iv) For the *model with a defect bond*:

(31) $$\begin{array}{ccc} Z_{C}^{\left(\mathrm{db}\right)}\left(N,K,K_{a},M\right) & = & {\left[\sqrt{2\sinh(2K)}\right]}^{N}\{\left[e^{K_{a}-K}-\frac{\sinh(K_{a}+K)}{\sinh(2K)}\right]D\left(N,M;e^{-4K}\right)\\ & & +\frac{1}{2}e^{K-K_{a}}\left[\frac{e^{2K_{a}}-e^{-2K}}{\sinh(2K)}\right]I\left(N,M,e^{-4K}\right)\}, \end{array}$$

where

(32) $$I\left(N,M,z\right):=\frac{4}{\pi }\int_{0}^{\pi /2}\cos\left(Mx\right)\;T_{N}\left(\frac{\cos(x)}{\sqrt{1-z}}\right)dx$$

and

(33) $$\begin{array}{c} D\left(N,M;z\right)=\frac{4}{\pi }\int_{0}^{\pi /2}\cos\left(Mx\right)\frac{\cos(x)}{\sqrt{1-z}}U_{N-1}\left(\frac{\cos(x)}{\sqrt{1-z}}\right)\;dx \end{array}$$

are defined in terms of the Chebyshev polynomials of the first $T_{N}\left(y\right)$ and second $U_{N}\left(y\right)$ kinds, respectively. As shown in [63,65], the above integrals can be expressed in terms of the Gauss hypergeometric functions. The results are

(34) I(N,M,z)=Nz(1−z)−N/2F1212(M−N+2),12(−M−N+2);2,z

and

(35) D(N,M,z)=(1−z)−N/2z{NF1212(M−N+2),12(−M−N+2);2,z+2(z−1−1)F1212(M−N+2),12(−M−N+2);1,z}.

From Equation (26), if we write M=mN and focus on the case where N≫1, we obtain the fluctuation-induced Helmholtz force $F_{H}^{\left(\mathrm{per}\right)}\left(K,m,N\right)$. Multiplying the result for $F_{H}^{\left(\zeta \right)}\left(K,m,N\right)$ by *N* provides us with the function $X_{H}^{\left(\zeta \right)}\left(K,m|N\right)$,

(36) $$X_{H}^{\left(\zeta \right)}\left(K,m|N\right)=NF_{H}^{\left(\zeta \right)}\left(K,m,N\right).$$

For the scaling behavior of $X_{H}^{\left(\zeta \right)}\left(K,m|N\right)$ close to T=0 in terms of $x_{t}=2Ne^{-2K}$, i.e., of the scaling combination $N/\xi_{t}$, with $\xi_{t}$ as the correlation length [111] in the vicinity of the zero temperature critical point, we have the following:

(i) In the case of *periodic* boundary conditions:

(37) $$X_{H}^{\left(\mathrm{per}\right)}\left(x_{t},m\right)=-\frac{1}{2}\sqrt{1-m^{2}}x_{t}+\frac{x_{t}I_{0}\left(\frac{1}{2}x_{t}\sqrt{1-m^{2}}\right)}{2\sqrt{1-m^{2}}I_{1}\left(\frac{1}{2}x_{t}\sqrt{1-m^{2}}\right)}-\frac{1+m^{2}}{1-m^{2}}$$

where $I_{0}$ and $I_{1}$ are the modified Bessel functions.

**Figure 5:**
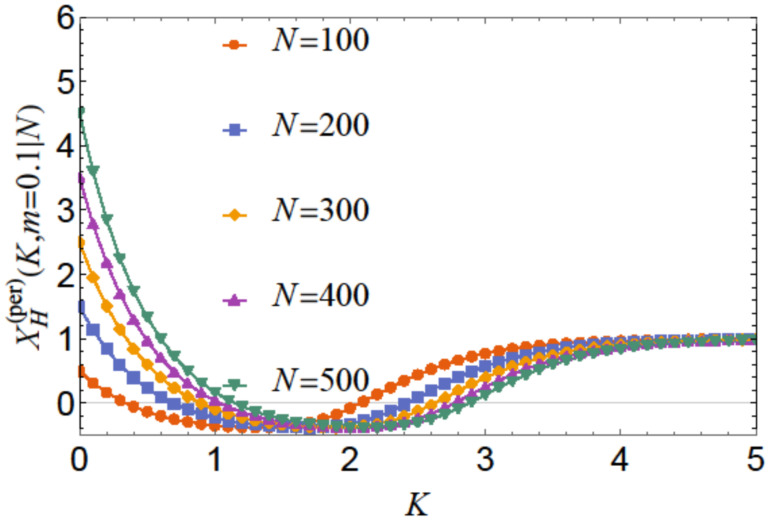
Behavior of the function $X_{H}^{\left(\mathrm{per}\right)}\left(K,m|N\right)$ (see Equation (36)) with N=100,200,300,400, and N=500. It can be observed that the function is *positive* for large and sufficiently small values of *K*, while being *negative* for relatively moderate values of *K irrespective* of the value of *N*. The larger the value of *N*, the stronger the repulsion for a small enough *K*; in the latter regime, the force is strongly repulsive irrespective of the value of *N*.

**Figure 6:**
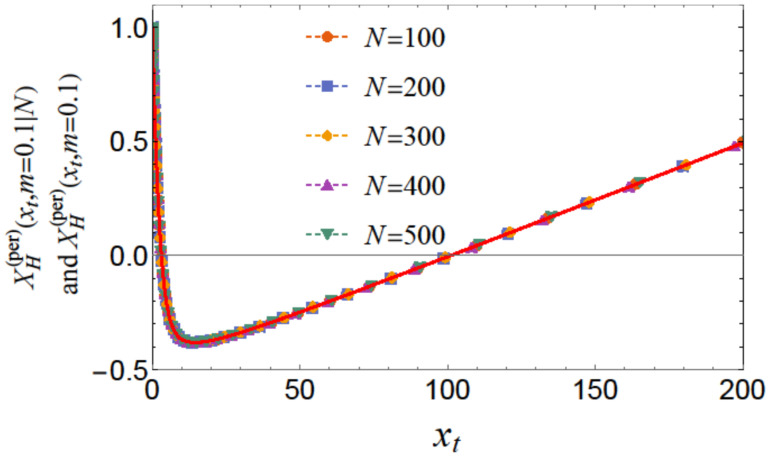
Behavior of the scaling function $X_{H}^{\left(\mathrm{per}\right)}\left(x_{t},m\right)$ for m=0.1. Inspection of the results obtained numerically from Equation (29) with N=100,200,300,400, and N=500 along with those from Equation (37) demonstrate perfect scaling and agreement. It can be observed that the function is *positive* for large values of $x_{t}$, *negative* for relatively moderate values of $x_{t}$, and again strongly repulsive for small values of $x_{t}$.

The behavior is shown in Figure 5 for m=0.1 and for N=100,200,300,400, and N=500. Focusing on the scaling regime (i.e., *K* and *N* are both large compared to 1). we end up with the *N*-independent scaling function $X^{\left(\mathrm{per}\right)}\left(x_{t},m\right)$. Figure 6 shows the behavior of this quantity as a function of $x_{t}$ for m=0.1.

**Figure 7:**
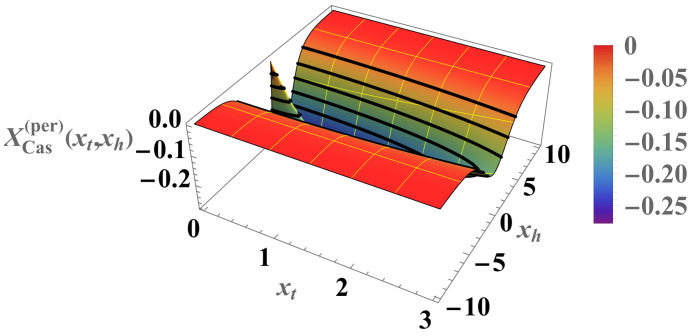
Relief plot of the scaling function $X_{\mathrm{Cas}}^{\left(\mathrm{per}\right)}\left(x_{t},x_{h}\right)<0$ for PBC (see Equation (38)). The function is always *negative*, corresponding to an *attractive* force symmetric about h=0.

**Figure 8:**
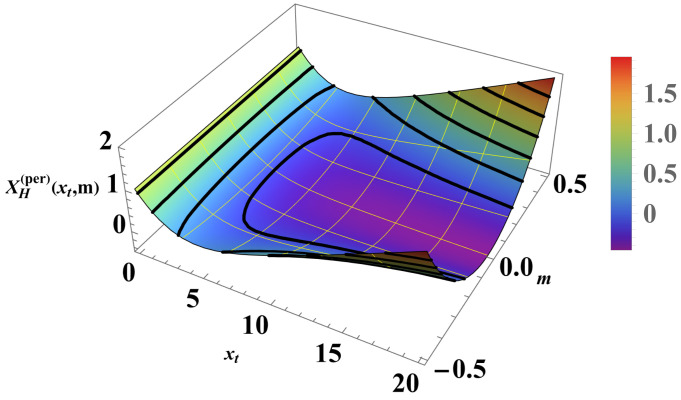
The figure shows the behavior of the function $X_{H}^{\left(\mathrm{per}\right)}\left(x_{t},m\right)$ for PBC; see Equation (37).

We can compare the above results with those for the Casimir force under the same boundary condition (see Figure 7 and Figure 8). For the Casimir force, with $x_{h}=N/\xi_{h}=2Nh$ we have

(38) $$\beta F_{\mathrm{Cas}}^{\left(\mathrm{per}\right)}\left(N,K,h\right)=\frac{1}{N}X_{\mathrm{Cas}}^{\left(\mathrm{per}\right)}\left(x_{t},x_{h}\right),\;\mathrm{where}\;X_{\mathrm{Cas}}^{\left(\mathrm{per}\right)}\left(x_{t},x_{h}\right)\;=\;-\sqrt{x_{h}^{2}+x_{t}^{2}}\;\frac{\exp\left[-\sqrt{x_{h}^{2}+x_{t}^{2}}\right]}{1+\exp\left[-\sqrt{x_{h}^{2}+x_{t}^{2}}\right]}.$$

As can be seen, contrary to the Helmholtz force, the Casimir force preserves its negative sign under periodic boundary conditions for *all* values of $x_{t}$ and $x_{h}$.

(ii) In the case of *antiperiodic* boundary conditions:

For the Helmholtz force, the corresponding result is

(39) $$X_{H}^{\left(\mathrm{anti}\right)}\left(x_{t},m\right)=\frac{1}{2}\frac{x_{t}I_{1}\left(\sqrt{1-m^{2}}x_{t}\right)}{\sqrt{1-m^{2}}I_{0}\left(\sqrt{1-m^{2}}x_{t}\right)}-\frac{1}{2}\sqrt{1-m^{2}}x_{t},$$

while for the Casimir force we have

(40) $$\beta F_{\mathrm{Cas}}^{\left(\mathrm{anti}\right)}\left(N,K,h\right)=\frac{1}{N}X_{\mathrm{Cas}}^{\left(\mathrm{anti}\right)}\left(x_{t},x_{h}\right),\;\mathrm{where}\;X_{\mathrm{Cas}}^{\left(\mathrm{anti}\right)}\left(x_{t},x_{h}\right)=\sqrt{x_{h}^{2}+x_{t}^{2}}\frac{\exp\left[-\sqrt{x_{h}^{2}+x_{t}^{2}}\right]}{1-\exp\left[\sqrt{x_{h}^{2}+x_{t}^{2}}\right]}>0.$$

**Figure 9:**
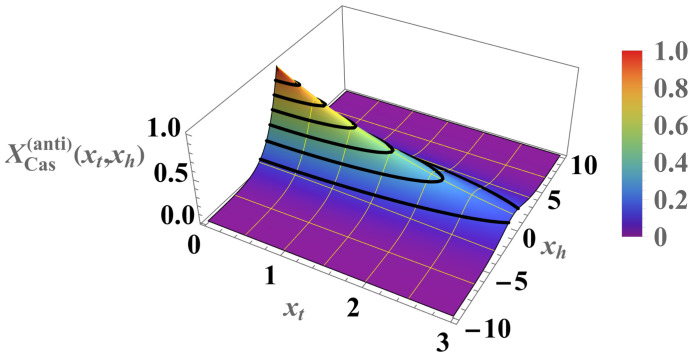
Relief plot of the scaling function $X_{\mathrm{Cas}}^{\left(\mathrm{anti}\right)}\left(x_{t},m\right)>0$ for ABC (see Equation (40)). Contrary to the periodic case, the force, is always *repulsive*.

**Figure 10:**
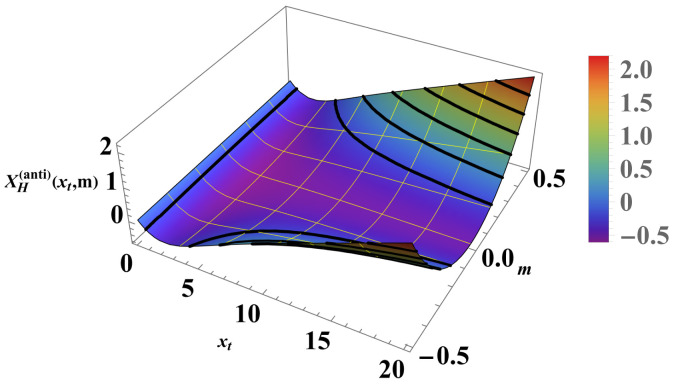
The figure shows the behavior of $X_{H}^{\left(\mathrm{anti}\right)}\left(x_{t},m\right)$ for ABC; see Equation (39).

Again, contrary to the Helmholtz force, the Casimir force preserves its positive sign under antiperiodic boundary conditions for *all* values of $x_{t}$ and $x_{h}$. A detailed comparison is shown in Figure 9 and Figure 10.

(iii) In the case of *Dirichlet–Dirichlet* boundary conditions:

For the Helmholtz force, the result is

(41) $$\begin{array}{cc} \; & X_{H}^{\left(D\right)}\left(x_{t},m\right)=\\ \; & \frac{\left[\left(1-m^{2}\right)\left(1-\sqrt{1-m^{2}}\right)x_{t}-2\left(m^{2}+1\right)\right]I_{1}\left(\frac{1}{2}\sqrt{1-m^{2}}x_{t}\right)+x_{t}\left(1-{\left(1-m^{2}\right)}^{3/2}\right)\sqrt{1-m^{2}}I_{0}\left(\frac{1}{2}\sqrt{1-m^{2}}x_{t}\right)}{2\left(1-m^{2}\right)\left[I_{1}\left(\frac{1}{2}\sqrt{1-m^{2}}x_{t}\right)+\sqrt{1-m^{2}}I_{0}\left(\frac{1}{2}\sqrt{1-m^{2}}x_{t}\right)\right]}, \end{array}$$

while for the Casimir force we derive

(42) $$X_{\mathrm{Cas}}^{\left(D\right)}\left(x_{t},x_{h}\right)=-\frac{\sqrt{x_{h}^{2}+x_{t}^{2}}}{r\left(x_{t},x_{h}\right)\exp\left(\sqrt{x_{h}^{2}+x_{t}^{2}}\right)+1},\;\mathrm{where}\;r\left(x_{t},x_{h}\right)=\frac{\sqrt{x_{h}^{2}+x_{t}^{2}}+x_{t}}{\sqrt{x_{h}^{2}+x_{t}^{2}}-x_{t}}.$$

**Figure 11:**
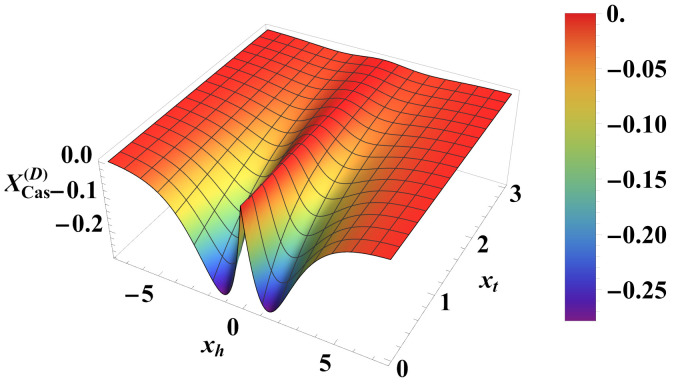
Behavior of the scaling function $X_{\mathrm{Cas}}^{\left(D\right)}\left(x_{t},x_{h}\right)$ of the Casimir force as a function of the scaling variables $x_{t}$ and $x_{h}$. It can be observed that the function is *negative* for *all* values of $x_{t}$ and $x_{h}$.

**Figure 12:**
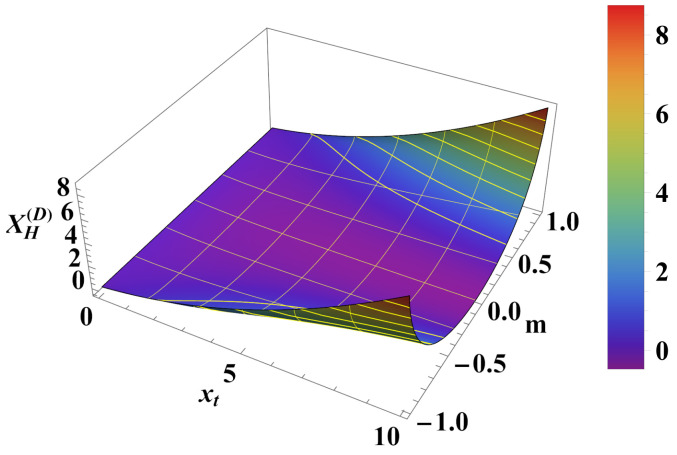
Behavior of the function $X_{H}^{\left(D\right)}\left(x_{t},m\right)$. One observes that the force can be both attractive *and* repulsive depending on the values of $x_{t}$ and *m*.

The comparison between the corresponding scaling functions is shown in Figure 11 and Figure 12. We conclude that the Helmholtz force under Dirichlet–Dirichlet boundary conditions shows behavior remarkably different from that of the Casimir force, which is always attractive for the same boundary conditions.

(iv) Ising chain with a *defect bond*:

For the Helmholtz force, the corresponding result is somewhat cumbersome:

(43) $$\begin{array}{cc} \; & X_{H}^{\left(db\right)}\left(x_{t},m\right)=-\frac{1}{2}\sqrt{1-m^{2}}\;x_{t}+\\ \; & x_{t}\frac{\frac{1}{4}e^{K_{a}}\left[I_{0}\left(\frac{1}{2}\sqrt{1-m^{2}}x_{t}\right)+I_{2}\left(\frac{1}{2}\sqrt{1-m^{2}}x_{t}\right)\right]+\frac{1}{2}e^{-K_{a}}\sqrt{1-m^{2}}I_{1}\left(\frac{1}{2}\sqrt{1-m^{2}}x_{t}\right)}{e^{-K_{a}}I_{0}\left(\frac{1}{2}\sqrt{1-m^{2}}x_{t}\right)+e^{K_{a}}I_{1}\left(\frac{1}{2}\sqrt{1-m^{2}}x_{t}\right)/\sqrt{1-m^{2}}}\\ \; & +m^{2}\frac{2\left[\left(m^{2}-1\right)x_{t}+2\right]I_{1}\left(\frac{1}{2}\sqrt{1-m^{2}}x_{t}\right)-\sqrt{1-m^{2}}x_{t}I_{2}\left(\frac{1}{2}\sqrt{1-m^{2}}x_{t}\right)-\sqrt{1-m^{2}}x_{t}I_{0}\left(\frac{1}{2}\sqrt{1-m^{2}}x_{t}\right)}{4\left(m^{2}-1\right)\left[I_{1}\left(\frac{1}{2}\sqrt{1-m^{2}}x_{t}\right)+\sqrt{1-m^{2}}I_{0}\left(\frac{1}{2}\sqrt{1-m^{2}}x_{t}\right)\right]}. \end{array}$$

For the scaling function of the Casimir force $X_{\mathrm{Cas}}\left(x_{t},x_{a},x_{h}\right)$, in this case we have

(44) $$\beta F_{\mathrm{Cas}}^{\left(\mathrm{db}\right)}\left(N,K,h\right)=\frac{1}{N}X_{\mathrm{Cas}}^{\left(\mathrm{db}\right)}\left(x_{t},K_{a},x_{h}\right)\;\mathrm{with}\;X_{\mathrm{Cas}}^{\left(\mathrm{db}\right)}\left(x_{t},K_{a},x_{h}\right)=-\frac{\sqrt{x_{h}^{2}+x_{t}^{2}}}{r\left(x_{t},K_{a},x_{h}\right)\exp\left(\sqrt{x_{h}^{2}+x_{t}^{2}}\right)+1},$$

where

(45) $$r\left(x_{t},K_{a},x_{h}\right)=\frac{\sqrt{x_{h}^{2}+x_{t}^{2}}+x_{t}\exp\left(-2K_{a}\right)}{\sqrt{x_{h}^{2}+x_{t}^{2}}-x_{t}\exp\left(-2K_{a}\right)}.$$

Obviously, if $r(x_{t},K_{a},x_{h})>0$, then we have $X_{\mathrm{Cas}}\left(x_{t},K_{a},x_{h}\right)<0$; furthermore, $X_{\mathrm{Cas}}\left(x_{t},K_{a},x_{h}\right)$ decays exponentially when $x_{h}^{2}+x_{t}^{2}\gg 1$.

**Figure 13:**
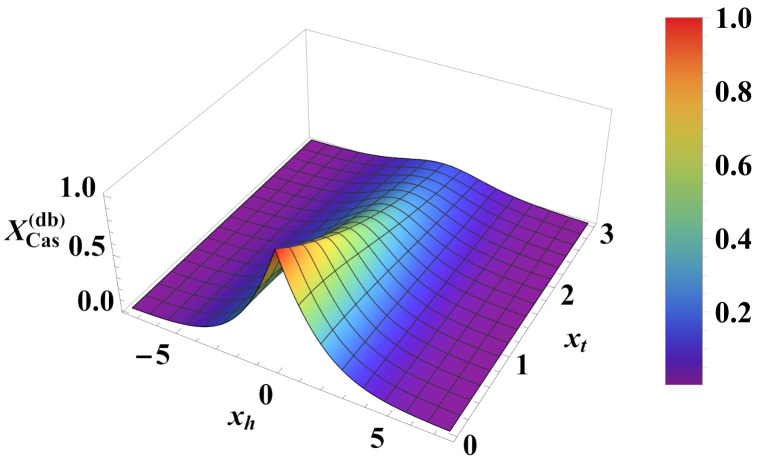
Behavior of the scaling function $X_{\mathrm{Cas}}^{\left(db\right)}\left(x_{t},x_{h}\right)$ of the Casimir force as a function of the scaling variables $x_{t}$ and $x_{h}$. It can be observed that the function *changes sign* for a negative $K_{a}=-3$ depending on the values of $x_{t}$ and $x_{h}$.

**Figure 14:**
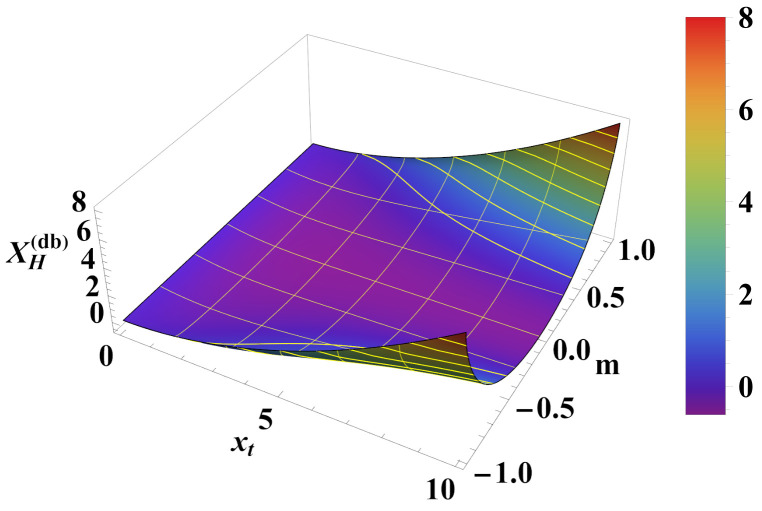
Behavior of the scaling function $X_{H}^{\left(db\right)}\left(x_{t},m\right)$ of the Helmholtz force. It can be observed that the force can be both attractive *and* repulsive depending on the values of $x_{t}$ and *m*.

A comparison of the behavior of the Helmholtz and Casimir forces for this particular case is shown in Figure 13 and Figure 14. It can be observed that in this case the Casimir force can *also* change sign; this happens for *negative* values of $K_{a}$, i.e., in the case where there are *competing interactions* within the chain.

### On the Connection between the Canonical and Grand Canonical Partition Functions

In the section, we briefly demonstrate that in the canonical ensemble the system possesses much more profound finite-size corrections with respect to the bulk behavior. For this, we use the the simplest case involving periodic boundary conditions as our example.

In the canonical ensemble, the partition function $Z_{\mathrm{CE}}\left(N,\beta ,M\right)$ can be written in the following form:

(46) $$Z_{\mathrm{CE}}\left(N,\beta ,M\right)=\sum_{\left\{s_{r}\right\}}\exp\left[-\beta H(\left\{s_{r}\right\})\right]\delta \left(\sum_{r}s_{r}-M\right).$$

Starting from Equation (46) and using the identity

(47) $$\delta \left(s\right)=\frac{1}{2\pi }\int_{-\infty }^{\infty }\exp\left[i\;s\;x\right]dx,$$

we consequently obtain

(48) $$\begin{array}{ccc} Z_{\mathrm{CE}}\left(N,\beta ,M\right) & = & \frac{1}{2\pi }\int_{-\infty }^{\infty }dx\sum_{\left\{s_{r}\right\}}\exp\left[-\beta H\left(\left\{s_{r}\right\}\right)+ix\left(\sum_{r}s_{r}-M\right)\right]\\ & = & \frac{1}{2\pi }\int_{-\infty }^{\infty }dx\exp\left[-i\;M\;x\right]\sum_{\left\{s_{r}\right\}}\exp\left[-\beta H\left(\left\{s_{r}\right\}\right)+ix\sum_{r}s_{r}\right]\\ & = & \frac{1}{2\pi }\int_{-\infty }^{\infty }dx\exp\left[-i\;M\;x\right]\;Z_{\mathrm{GCE}}\left(N,\beta ,i\;x\right). \end{array}$$

Thus,

(49) $$Z_{\mathrm{CE}}\left(N,\beta ,M\right)=\frac{1}{2\pi }\int_{-\infty }^{\infty }dx\exp\left[-i\;M\;x\right]\;Z_{\mathrm{GCE}}\left(N,\beta ,i\;x\right).$$

Using the definitions of the Gibbs free energy density f(β,h,N)

(50) $$Z_{\mathrm{GCE}}\left(N,\beta ,h\right)\equiv \exp\left[-N\beta f\left(N,\beta ,h\right)\right]$$

and Helmholtz free energy

(51) $$Z_{\mathrm{CE}}\left(N,\beta ,M\right)\equiv \exp\left[-N\beta a\left(N,\beta ,h\right)\right],$$

and introducing the magnetization per particle m=M/N, we can rewrite Equation (49) in the following form:

(52) $$\exp\left[-\beta a\left(N,\beta ,m\right)\right]=\frac{1}{2\pi }\int_{-\infty }^{\infty }dx\exp\left\{-N\left[\beta f\left(N,\beta ,\;ix\right)+i\;m\;x\right]\right\}.$$

For N≫1, integrals of this kind can be estimated using the saddle point method (see [112,113]), which in this case reads

(53) $$\int_{R}dx\exp\left[-Ng\left(x\right)\right]≃\exp\left[-Ng\left(x_{0}\right)\right]\sqrt{\frac{2\pi }{Ng^{\prime \prime }\left(x_{0}\right)}}\left(1+O(N^{-2})\right);g^{\prime }\left(x_{0}\right)=0.$$

With the interpretation $ix_{0}\equiv h$, we obtain

(54) $$\exp\left[-N\beta a\left(N,\beta ,m\right)\right]≃\exp\left[-N\left(\beta f(N,\beta ,h)+mh\right)\right]\sqrt{\frac{2\pi }{N\beta \chi (N,\beta ,h)}}\left(1+O(N^{-2})\right),$$

where the extreme condition

(55) $$\frac{\partial }{\partial x}\left[\beta f\left(N,\beta ,i\;x\right)+i\;mx\right]=i\;[m+\frac{\partial }{\partial h}\left[\beta f\left(N,\beta ,h\right)\right]=0$$

leads to the standard statistical–mechanical relation m=−∂[βf(N,β,h)]/∂h and $\chi \left(N,\beta ,h\right)\equiv -\partial^{2}f/\partial h^{2}$ is the susceptibility of the finite system in the grand canonical ensemble. Obviously, Equation (54) can be rewritten as

(56) $$\beta a\left(N,\beta ,m\right)=\beta f\left(N,\beta ,h\right)+mh-\frac{1}{2N}\ln\frac{2\pi }{N\beta \chi (N,\beta ,h)}.$$

Equation (56) implies that the leading finite-size corrections in the Helmholtz free energy are on the order of lnN/N. These are much stronger than for the Gibbs free energy, for which they are well known to be exponentially small in *N* away from the critical temperature [57,59].

Taking the limits $\lim_{N\to \infty }f\left(N,\beta ,h\right)=f_{b}\left(\beta ,h\right)$ and $\lim_{N\to \infty }a\left(N,\beta ,m\right)=a_{b}\left(\beta ,m\right)$ on the right-hand side of Equation (56), we arrive at the Legendre transformation between the two ensembles, as known from standard thermodynamics:

(57) $$a_{b}\left(\beta ,m\right)=f_{b}\left(\beta ,h\right)+hm.$$

Note that the relation provided by Equation (49) can be inverted, which leads to

(58) $$Z_{\mathrm{GCE}}\left(N,\beta ,iy\right)=\int_{-\infty }^{\infty }dM\exp\left[i\;M\;y\right]\;Z_{\mathrm{CE}}\left(N,\beta ,M\right),$$

or with h=iy, to the self-explained relation

(59) $$Z_{\mathrm{GCE}}\left(N,\beta ,h\right)=\int_{-\infty }^{\infty }dM\exp\left[h\;M\right]\;Z_{\mathrm{CE}}\left(N,\beta ,M\right)=\int_{-N}^{N}dM\exp\left[h\;M\right]\;Z_{\mathrm{CE}}\left(N,\beta ,M\right),$$

where we have taken into account that |M|≤N. As Equation (59) implies, the partition functions $Z_{\mathrm{GCE}}\left(N,\beta ,h\right)$ and $Z_{\mathrm{CE}}\left(N,\beta ,M\right)$ are mutually related through an integral transformation; however, their finite-size behavior is different. Because of this, it is reasonable to expect (as turns out to be the case for the Ising model) that, for a given ensemble, the FIF are strongly *ensemble-dependent*.

## 6. Concluding Comments and Discussion

The interest in fluctuation-induced phenomena has blossomed in recent years due to their importance in the rapidly developing field of nanotechnology, where the van der Waals force (vdWF) and QED CF play a dominant role between neutral nonmagnetic objects below a micrometer distances. The implies that these forces play a key role in micro- and nanoelectromechanical systems (MEMS/NEMS) [114,115,116] operating at such distances. In vacuum or gas medium, they lead to irreversible and usually undesirable phenomena such as stiction (i.e., irreversible adhesion) and pull-in due to mechanical instabilities [117,118,119]. Indeed, being negligible at macroscopic distances, the Casimir force can become impressively strong at the micro- and nanoscales. According to Equation (2), if two perfectly conducting parallel metal plates are facing each other at a distance on the order of 10 nm in vacuum and at zero temperature, the attractive Casimir force per area, i.e., the Casimir pressure, can be as large as one atmosphere! Such a large force strongly influences the performance of micro- and nanomachines by causing stiction, in which their moving parts stick together (de facto irreversibly) and stop working. Naturally, this affects the design and functioning of devices at these scales. This can be considered as the *first fundamental problem of nanotechnology*.

Closely related to the above is another troubling effect: when a particle’s characteristic size is scaled down below a micrometer (say, a colloid particle), then the role of its weight becomes negligible. As a result, when one tries to release such a neutral particle from, say, the surface of whatever handling device used in air or vacuum, the particle will not fall under the force of gravity, instead sticking to the surface due to the effect of the omnipresent vdWF. If one charges the particle in an attempt to release the particle, then this causes vibration on the part of the surface in question, meaning that the released particle might move in an uncontrollable way, potentially even leaving the observation field of the apparatus controlling the performance of the operation. This is the main reason why the handling, feeding, trapping, and fixing of micro- and/or nanoparticles remains the main bottleneck in micromanufacturing, and is far from being solved in a satisfactory fashion [120]. This issue can be considered as the *second fundamental problem of nanotechnology*. It should be noted that when the “particle” is further scaled down to the point of atomic, molecular, or nanoparticle-scale interaction with a macrobody, one encounters the so-called fluctuation-induced Casimir–Polder forces, where the interaction is proportional to the polarizability of the particle [121].

Thus, formalizing the above, one of the main problems in micro- and nanoassembly is the precise and reliable manipulation of a micro- or nanoparticles. This includes moving the particle from some starting point where it is to be taken from to some end point where it is to be placed. In this respect, it would seem ideal to modify the net force between the manipulated particle and the operating device (sometimes called the gripper) in such a way as to make it repulsive at short distances between the handling surfaces and the particle and attractive at larger ones. It is clear that the ability to modify the Casimir interaction can strongly influence the development of MEMS/NEMS. However, several theorems seriously limit the possible search for repulsive QED CF [122,123,124]. Currently, apart from some suggestions for achieving QED Casimir repulsion in systems that are out of equilibrium, the only way to obtain such a repulsive force that has been well verified experimentally is to characterize the interaction between two different materials by dielectric permittivities $\varepsilon^{\left(1\right)}$ and $\varepsilon^{\left(2\right)}$, separated by a fluid with permittivity $\varepsilon^{\left(0\right)}$ [66,77,125] such that Equation (10) is fulfilled in a sufficiently broad frequency range. In [82,126,127,128,129,130], QED Casimir repulsion has already been observed experimentally for a sphere–plate geometry. In order to minimize the potential negative effects of all possible circuitry at such small distances, along with complications involving isolation and possible problems involving chemical reactions, it seems that one promising strategy for overcoming the obstacles mentioned above is to choose a fluid as a medium that possesses no free changes dissolved in it, that is inert, and that does not interact chemically with the materials. This leads to the choice of a fluid such as a nonpolar liquefied noble gas that has critical parameters as close as possible to the normal ones. Such a strategy for overcoming the difficulties described above has been suggested in [131,132].

Currently, several issues related to the QED Casimir effect remain to be resolved:

- Additional interest in the QED Casimir effect is being driven by certain theoretical predictions stemming from attempts to construct unified field theories of the fundamental forces. According to these predictions, Newton’s gravitational law might be modified at sub-millimeter distances. By measuring the Casimir force and comparing these data with the predictions of theory, it could be possible to obtain, inter alia, constraints for the parameters characterizing Yukawa-type deviations from Newtonian gravity, which could help to test the validity of such ideas [133,134,135].
- There has been speculation about possible relations of the Casimir effect to topics such as dark matter and cosmology [28,136,137,138,139]. These relations are linked to discussions about the physical meaning of the zero-point energy of quantum fields, the cosmological constant problem, and the physical interpretation of the Casimir effect. There is a considerable body of literature dealing with the physical source of the Casimir force. An extensive discussion of this issue can be found in [7,13], and more recently in [28,137,138,140].
- It has been suggested [141,142] that hypothetical chameleon interactions, which might explain the mechanisms behind dark energy, might be detected through high-precision force measurements. In [142,143], the authors proposed the design, fabrication, and characterization of such a force sensor for chameleon and Casimir force experiments using a parallel-plate configuration. The idea is to measure the total force between two parallel plates as a function of the density of a neutral gas allowed into the cavity. As the density of the gas increases, the mass of the chameleon field in the cavity increases, giving rise to a screening effect of the chameleon interaction.
- Regarding the description of the dielectric properties, especially for the thermal contribution to the Casimir effect, there has been discussion of this topic over the course of more than two decades [144]. We would just mention that the disagreement between theoretical predictions and precise experimental results has placed the focus on the proper account of dissipation in the description of the material optical response; in several experiments on the QED effect between metallic objects, a simple non-dissipative model has provided the best description. The same experiments appear to exclude the account of dissipation provided by the commonly used Drude model [95,145,146]. An additional issue involves the description of the role of free electrons in semiconductors [147], where attempts to date have not reached a unanimous consensus.

As far as the thermodynamic Casimir effect has been investigated within the framework of statistical mechanics, most results belong to classical systems in the grand canonical ensemble. It is expected that in the future there will be attempts to extend these results to dynamical systems, quantum systems (including systems with different types of quenches), systems described by other (say) canonical or micro-canonical ensembles, and systems exhibiting disorder and topological phase transitions as well as their combination. With regard to the example of the Ising model, we have outlined the grand canonical ensemble in Section 4.1. We have also pointed out that it is possible to consider ensemble-dependent fluctuation-induced forces; for example, we have outlined the canonical ensemble in Section 4.3 and the definition of the corresponding fluctuation-induced force in Section 4.4. There, following [62,63,64,65], we have shown that these forces have behaviors that are quite different from that of the Casimir force under the same boundary conditions and under the same geometry (see Figure 5, Figure 6, Figure 8, Figure 10, Figure 12 and Figure 14). We would note that all of the issues studied for the Casimir forces are objects of investigation in, say, canonical or micro canonical ensembles as well.

In order to avoid providing the incorrect impression that the FIFs considered in this short review are the only ones, we briefly mention a few other FIFs which, due to space limitations, we were not able to provide further details on:

***(i)*** For fluctuation-induced forces related to charge fluctuations, see [148,149,150,151,152,153,154,155].

***(ii)*** For fluctuation-induced forces between objects on a fluctuating membrane or on fluid interfaces, see [156,157,158,159,160,161,162].

***(iii)*** For the phonon Casimir effect due to phonon-mediated interaction between defects in condensed matter systems, see [163,164].

***(iv)*** There is also a so-called non-equilibrium thermodynamic (hydrodynamic) Casimir-like effect, where correlations in fluids in nonequilibrium or nonequilibrium steady states are of importance; for this, see [165,166,167,168,169,170,171,172].

***(v)*** Fluctuation-induced Casimir forces in granular fluids have been reported in [173].

***(vi)*** In nematic liquid crystals, the fluctuations of the nematic director are responsible for the long-range nature of the corresponding Casimir force [98,174,175,176,177,178,179,180].

***(vii)*** Studies of the Casimir effect in active matter systems include [172,181,182,183,184,185].

***(viii)*** When the boundary conditions are time-dependent, the change in the vacuum energy leads to the so-called *dynamical* Casimir effect; see [39,40,41,42,43,44,45,46].

It it worth mentioning that the bodies *A* and *B* discussed above can also be in motion with respect to the medium *C* or to each other; these options generate a plethora of possible FIFs. In addition, because FIFs depend on the geometry of the system, there is abundance of geometry-dependent phenomena.

We close this list of references by pointing to the earliest consideration of FIF that we are aware of, that of Einstein [186]; as early as 1907, he considered voltage fluctuations in capacitor systems due to a nonzero temperature *T*. Similar effects are known to occur in wires [187,188]. For example, the famous Johnson–Nyquist formula describes the dependence of the mean square noise current $\langle I^{2}\rangle$ on the resistivity *R* and temperature *T* of a resistor according to $\langle I^{2}\rangle =4k_{B}\;T\Delta f/R$, where Δf is the measurement bandwidth. Such fluctuations lead to forces which are of serious interest in the operation of electromechanical devices [189] downscaled to the micro- or nanoscale level.

Finally, we finish this short review by noting that FIFs are not only a topic of interest for academic investigations; for both the QED Casimir effect (for which the first practical applications are currently under discussion; see, e.g., [34,37,54,55,190,191,192,193,194,195] and references therein) and the CCE (see, e.g., refs. [196,197,198,199,200,201,202,203,204,205,206]), a number of applications have already been considered. We specifically mention [207], which describes how to use the CCF to manipulate the position and orientation of nanoparticles.

## Abbreviations

The following abbreviations are used in this manuscript:

CCF	Critical Casimir Force
QED	Quantum Electrodynamics
DA	Derjaguin Approximation
CF	Casimir Force
MEMS/NEMS	Micro/Nanoelectromechanical Systems
CC	Critical Casimir
HF	Helmholtz Force
CCE	Critical Casimir Effect
FIF	Fluctuation-Induced Force
GCE	Grand Canonical Ensemble
PBC	Periodic Boundary Conditions.
